# The role of dendritic cells in COVID-19 infection

**DOI:** 10.1080/22221751.2023.2195019

**Published:** 2023-05-09

**Authors:** Xuying Wang, Fei Guan, Heather Miller, Maria G Byazrova, Fabio Candotti, Kamel Benlagha, Niels Olsen Saraiva Camara, Jiahui Lei, Alexander Filatov, Chaohong Liu

**Affiliations:** a Department of Pathogen Biology, School of Basic Medicine, Tongji Medical College, Huazhong University of Science Technology, Wuhan, People’s Republic of China; b Tongji Hospital Affiliated to Tongji Medical College, Huazhong University of Science and Technology, Wuhan, People’s Republic of China; c Cytek Biosciences, R&D Clinical Reagents, Fremont, CA, USA; d Laboratory of Immunochemistry, National Research Center Institute of Immunology, Federal Medical Biological Agency of Russia, Moscow, Russia; e Division of Immunology and Allergy, Lausanne University Hospital and University of Lausanne, Lausanne, Switzerland; f Institut de Recherche Saint-Louis, Université de Paris, Paris, France; g Laboratory of Human Immunology, Department of Immunology, Institute of Biomedical Sciences, University of São Paulo (USP), São Paulo, Brazil

**Keywords:** SARS-CoV-2, COVID-19, dendritic cell, immunopathology, viral infection

## Abstract

The persistent pandemic of coronavirus disease in 2019 (COVID-19) caused by severe acute respiratory syndrome coronavirus 2 (SARS-CoV-2) currently poses a major infectious threat to public health around the world. COVID-19 is an infectious disease characterized by strong induction of inflammatory cytokines, progressive lung inflammation, and potential multiple organs dysfunction. SARS-CoV-2 infection is closely related to the innate immune system and adaptive immune system. Dendritic cells (DCs), as a “bridge” connecting innate immunity and adaptive immunity, play many important roles in viral diseases. In this review, we will pay special attention to the possible mechanism of dendritic cells in human viral transmission and clinical progression of diseases, as well as the reduction and dysfunction of DCs in severe SARS-CoV-2 infection, so as to understand the mechanism and immunological characteristics of SARS-CoV-2 infection.

## Introduction

In December 2019, an outbreak of pneumonia of unknown origin attracted domestic and international attention. on 7 January 2020, Chinese scientists extracted and isolated a novel coronavirus (CoV) from pneumonia patients, named 2019-nCoV [1]. It is highly infectious and rapidly spreads all over the world [2]. In March 2020, according to the development trend of the epidemic situation and the assessment of the number of patients and deaths, WHO declared COVID-19 a pandemic [3]. Since the 31st of December 2019 and as of early 2022, 328,558,243 cases of COVID-19 have been reported, including 5,548,696 deaths. COVID-19 patients have symptoms of respiratory diseases, like cough, shortness of breath, sore throat, and fever with other symptoms. Most patients are mildly symptomatic or even asymptomatic, but there are also serious patients who develop severe pneumonia and death [4]. The organism's immune system, the intensity of virus replication, and the degree of tissue and organ damage determine the severity of the disease in infected people. Although the pulmonary inflammation caused by SARS-CoV-2 has attracted extensive attention all over the world in recent years, and scientists have also carried out relevant researchs, the pathology and pathogenesis of the disease are not entirely known [5,6]. As a significant part of the human immune system, dendritic cells (DCs) are also very important in viral infection, but their role and function in SARS-CoV-2 infection are not completely distinct.

DCs have an important role in generating immune responses, linking innate and adaptive immunity. It is considered to be the most efficient antigen-presenting cells (APCs). The newborn dendritic cells are derived from CD34+ hematopoietic stem cells (HSCs) in the bone marrow. Immature DCs are located in the skin and mucous membranes on the body surface, such as the gastrointestinal tract, respiratory tract, lung and urogenital tract [7]. When the pathogen invades the body, it contacts the immature dendritic cells on the surface of the body's mucosa, and the immune response mediated by dendritic cells begins to start. Immature dendritic cells recognize, process and present pathogen antigens and produce cytokines under the action of DAMP (damage-associated molecular pattern) and PAMP (pathogen-associated molecular pattern) [8]. During the process from immature to mature, DCs migrate to lymphoid tissue and activate immature B and T lymphocytes. DCs also interact with innate immune cells, such as monocyte macrophages and NK cells, so as to assist in regulating innate immune response. As mentioned above, if the maturation process of DCs cannot be completed successfully, it will directly affect the initiation of adaptive immunity and the progress of innate immunity, thus consequently affecting the clearance of pathogens.

The phenotype and function of maturing DCs are becoming increasingly understood. Immature DCs express a low level of costimulatory molecules and major histocompatibility complexes (MHCs). Immature DCs are primarily situated in organismic barrier tissues, for example the skin, lymphoid tissues and mucous membrane, and function for antigen uptaking and processing. DCs mature during bodily injury, inflammation, microbial invasion, or tumours growth. After maturation, the endocytic ability of DCs will diminish, while the ability to produce immunostimulatory cytokines will be enhanced, which act to regulate the activity of innate immune cells. In addition, the migration of mature DCs to lymphoid tissues is enhanced and they are presented to CD8+ and CD4+ T cells by efficient antigen presentation to drive adaptive immune response.

**Figure 1.**
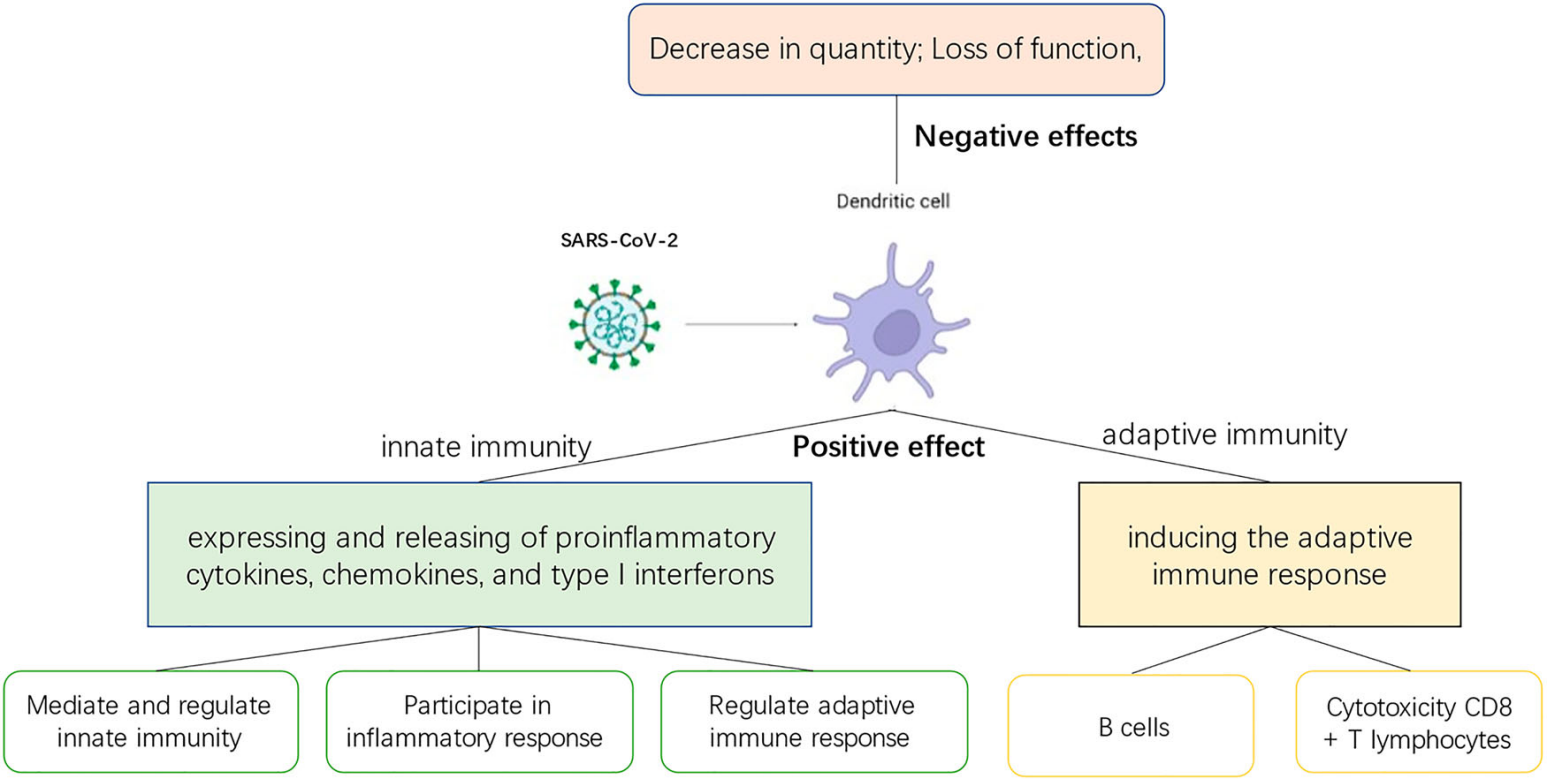
The role of dendritic cells in COVID-19 infection. When dendritic cells come in contact with viruses, they are stimulated and play different roles. Positive effect: It means that dendritic cells exert immune functions and play an important role in the body's resistance to viral invasion, connecting innate and specific immunity. Negative effect: It refers to the fact that viral infection of dendritic cells leads to decrease in the number and loss of function of dendritic cells, which is detrimental to the elimination of the invading virus by the organism.

This review focuses on the role of dendritic cells in innate and adaptive immunity against viruses and their role in the transmission of SARS-CoV-2. The purpose of this paper is to conceptualize the role of DCs in the pathogenesis and immune mechanisms of COVID-19, and the potential value of DCs in therapy [9] (Figure 1).

## General information of SARS-CoV-2

SARS-CoV-2 belongs to coronaviruses, which are a group of enveloped positive single stranded RNA genome viruses. According to previous epidemiological history, severe respiratory diseases such as coronavirus middle respiratory syndrome (MERS) and severe acute respiratory syndrome (SARS) were reported [10]. The genome size of SARS-CoV-2 is determined to be approximately 30 kb and contains 14 open reading frames [11]. The positive single stranded RNA genome encodes the structural proteins (S protein, E protein, M protein, and N protein) along with 16 non-structural proteins (NSPs). NSPs are responsible for the replication and transcription of genes, while structural proteins are responsible for the assembly of new virions [12].

The process of virus entering cells is closely related to S protein. This protein contains S1 subunit and S2 subunit. The role of S1 subunit is to specifically combine with the host cell receptor angiotensin converting enzyme 2 (ACE2), while S2 subunit is used to mediate the fusion of virus and host cell membrane [13]. SARS-CoV-2 gets into the host cells through ACE2 receptor expressed on the host cell membrane [14]. The receptor mainly exists in the epithelial cell membrane of lung, mucosa and small intestines. Because of the receptor location, person to person transmission tends to depend on aerosol droplets from the infected person. The S1/S2 site can be cleaved by the transmembrane serine protease 2 (TMPRSS2) via the plasma membrane pathway or by the internal lysosomal cathepsin L mediating viral cell membrane fusion via the endosomal pathway, thus allowing efficient entry of the virus into the cell [15]. After getting into cells, the RNA single stranded genome triumphantly entry the cell, where it generates replicase proteins that can carry out the next activity. The translated macromolecular protein is encoded by the virus, and the translated protease is hydrolysed into a single replicase complex nonstructural proteins (NSPs). The replication of single positive strand RNA begins with virus induced endoplasmic reticulum double membrane vesicles (DMV). The positive strand genome entering the double membrane vesicles of endoplasmic reticulum is replicated as the template of full-length negative strand RNA and sub genome (SG) RNA. In the next step, the structural protein and helper protein produced by sgRNA translation will be inserted into the endoplasmic reticulum Golgi intermediate region (ERGIC) for virus particle assembly. Subsequently, the single positive strand RNA genome is inserted into the newly synthesized virus particles to form a new virion with infectious ability. These virus particles are secreted by the plasma membrane.

The reported patients with COVID-19 have variable severity of clinical symptoms. The main symptoms of respiratory tract include fever, chest tightness, cough and shortness of breath; other symptoms such as nasal congestion, rhinorrhea and diarrhoea can also occur. Severely ill patients suffer from hypoxia, shortness of breath, sweating and peripheral weakness. The lesions of critically ill patients may accumulate other tissues and organ systems, resulting in the damage of other systems and serious complications. Thus, the symptoms of the patients are diverse, but mainly respiratory symptoms as the main manifestations. We cannot rely on one symptom to determine whether it is a SARS-CoV-2 infection or not. To diagnose SARS-CoV-2 infection, we should rely on laboratory tests, such as nucleic acid test for SARS-CoV-2. According to the clinical symptoms and patients’ body reactions of 2019 coronavirus disease, the development process of the disease can be divided into three stages [16]: 1. phase I: asymptomatic phase, where inhaled virus binds to nasal epithelial cells and begins to replicate, proliferating locally and inducing a limited immune response; 2. phase II: downward migration of virus into the lower respiratory tract, triggering a strong immune response, leading to pathological changes of congestion, edema and inflammatory cell infiltration with obvious clinical manifestations of COVID-19 disease; 3. phase III: hypoxia and progression to acute pneumonia, SARS-CoV-2 reaches the functional or gas exchange units of the lungs, which in turn can progress to acute respiratory distress syndrome (ARDS) eventually leading to possible multi-organ failure. In humans, the immune response plays an important role in fighting SARS-CoV-2. The immune response can clear virus, inhibit viral replication, and promote tissue repair; it also plays a significant role in SARS related pathogenicity.

## The role of dendritic cells in COVID-19 infection

Dendritic cells (DCs) are bone marrow-derived cells that are distributed in the periphery as immature dendritic cells. In the respiratory system, DCs respond dynamically to inflammation produced by local tissues of the airways and lung [17]. DCs express abundant receptors, for the recognition of conserved pathogen patterns. When immature dendritic cells are activated by a pathogen, a maturation process is initiated, consisting of the secretion of cytokines, and the expression of surface molecules, such as MHC and costimulatory molecules. In turn, the morphology of DCs changes during this process with the reorganization of their cytoskeleton and the surface expression of some integrin and chemokine receptors. The expression of these molecules enables dendritic cells to obtain the ability of migration and costimulation. The S protein, particularly its receptor-binding domain (RBD) can induce the maturation and activation of dendritic cells. S protein and RBD increase the number of MHC class I and MHC class II molecules on dendritic cell membrane, up-regulate the expression of CD40 and CD80, while RBD also up-regulates the expression of CD86 [18]. Upregulation of costimulatory molecules marks the activation of DCs. Together, these findings indicate that maturation and activation of DCs are due to recognition of the S proteins and RBD.

There are three dendritic cell subtypes in human lung tissue: myeloid/conventional dendritic cell types 1 and 2 (referred to as cDC1 and cDC2 in mice) and plasma cell like dendritic cell (pDC) [18]. In human tissues, cDC1 are expressed as CD141+, and cDC2 are expressed as CD1c+. cDC1s are distributed in the vascular wall and mucosa, where they are important stimulating cells of Th1 response. There is a distribution of cDC2 in the lamina propria, where their function is to produce inflammatory chemokines required for the aggregation of different inflammatory cells at the lesion site. cDC2 may also be involved in the establishment of immune tolerance [18]. pDCs exist in the whole lung tissue, including airway, lung parenchyma, and lung stroma. pDCs play a significant role in innate immunity. Because it can produce a large amount of type I interferon. Except for these three dendritic cell subtypes, DC can also be produced by circulating monocytes during inflammation, namely mo-DCs [18,19]. The classification and functions of dendritic cells are shown in Table 1 [18,20–31].

**Table 1.** DC subtype and function.

DC subtype		Function
pre-DCs [20–23] 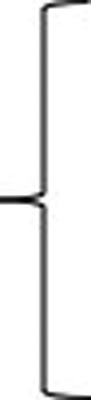	cDC1	cDC1s have a high intrinsic capacity to cross-present antigens, due to expression of the CLEC9A c-type lectin [24], and activate CD8+, Thl, and NK cells [25].
	cDC2	cDC2s express various pattern recognition receptors (PRRs) and can promote a wide range of immune responses, especially CD4+ T-cell responses [26].
	pDC	pDCs express high levels of endosomal nucleic acid sensing Toll-like receptors (TLRs) TLR7 and TLR9. pDCs respond to these nucleic acids with massive secretion of IFN-I and also produce type Ill interferon (IFN-λ or IL-28/lL-29) and additional cytokines (e.g. TNF-a) and chemokines [27–29]
	mo-DC	mo-DCs have a key role in inflammation [30] and infection [31], often termed “inflammatory mo-DC” [18].

DCs encompass a heterogeneous family of bone marrow-derived cells and are found in the peripheral blood, lymphoid organs, and tissues. According to the most recent DC classification, conventional DCs (cDCs) and plasmacytoid DCs (pDCs) are the two main functional DC subtypes. Conventional DCs encompass a new subset with monocyte-like characteristics, named monocyte-derived mo-DCs or DC3.

Although studies on other viral infectious diseases have proved that dendritic cells play an important role in the immunology of disease occurrence and development, we still cannot fully explain the role of dendritic cells in the progress of COVID-19. However, there is evidence that dendritic cells may be involved in the development and evolution of COVID-19.

### The role of dendritic cells in innate immune responses

Innate immunity is the defense system of human body against external injury and self-abnormality [32]. The innate immune response system forms a line of defense against conserved components of microorganisms, generating a rapid inflammatory response. Cells that make up the innate immune system include myeloid cells and innate lymphoid cells, which in turn include monocytes, macrophages, dendritic cells (DCs) and granulocytes. These cells enable direct clearance of pathogens and are able to turn on adaptive immune responses. DCs are significant members of the innate immune response and are involved in orchestrating the adaptive immunity.

Innate immune cells, including dendritic cells, utilize pathogen recognition receptors expressed on the cell membrane to bind and recognize pathogen associated molecular patterns. There are many different classes of pathogen recognition receptors expressed by innate immune cells, which are C-type lectin receptors, nod-like receptors (NLRs), RIG-I-like receptors (RLRs), and toll-like receptors (TLRs) [33,34]. The RNA genome contained in SARS-CoV-2 is recognized by RNA sensors located in the cytoplasm and endosomes, such as RIG-I and TLRs (TLR2, TLR3, and TLR7) [35–37]. RIG-I and TLRs afterwards activate interferon regulatory factor 3 (IRF3) and nuclear factor kappa-light-chain-enhancer of activated B cells (NF-κB), which enhances the transcription and translation of corresponding genes, inducing the expression and release of proinflammatory cytokines, chemokines, and type I interferons [38].

pDCs have an irreplaceable role in the organismic first line of defensing against viral replication, which relies on their ability to produce IFN-I via TLR-7/8 stimulation [39,40]. IFN-I can induce a strong antiviral response and limit virus replication in early stage of virus invasion [41]. pDCs are the important source of IFN-I, once activated, having an irreplaceable role in the early antiviral response [42,43]. Patients with acute SARS-CoV-2 infection exhibit defective DC numbers and reduced TLR9 dependent IFN-α production [44]. The occurrence of IFN-α is associated with the severity of physical damage caused by viral infection. IFN expression rises with disease severity in infected patients. Higher in patients with severe SARS-CoV-2 acute infection than in those with mild symptoms [44]. SARS-CoV-2 activated pDCs are able to efficiently produce type I and type III IFNs [45]. Pathogen recognition receptors (PRRS) combined viral nucleic acids, which rapidly trigger IFN-I production [46]. IFN-1 induced signals are aggregated to activate transcription factors. When transcription factors enter the nucleus, they rapidly induce hundreds of IFN stimulating genes (ISG) to express [41,47]. The waterfall effect of this signalling molecular cascade arises in any cell type exposed to IFN-I. And those downstream molecules (including proinflammatory cytokines) produced by ISGS and IFN-I control have powerful immune functions. They play such roles as directly inhibiting viral replication and activating various immune cells [48,49]. Therefore, the IFN-1 response is a part in first protective line of antiviral infection to facilitate viral removal, induce tissue repair and extend the adaptive immune response to the virus. SARS-CoV-2 induces IFN-I response less vigorously than other respiratory RNA viruses in vitro culture and animal models and appears to be a defective derivative of the IFN-I response [50,51]. Clinically, patients have lower than normal levels of IFN-I in serum, even though the expression of ISGS is still detectable [52–54]. Thus, limited IFN-I generation is sufficient to induce ISG. Perhaps the production of IFN-1 may be limited to specific immune cells, such as pDCs. SARS-CoV-2-derived IFN-I was produced in smaller amount than SARS [53], and SARS itself is a low elicitor in organism [55–57].

The production of interferon by DCs can lead to the production of CCL3 (MIP-1α) [58,59]. Recruiting NK cells, directly kill virus infected cells and release IFN-γ [60].

### The role of dendritic cells in adaptive immune responses

Innate immune system provides a basic mechanism for rapid detection and removal of viruses, but the adaptive immune response is necessary to effectively remove many viruses and establish immune memory. The adaptive immune system provides two methods of immunity antibodies from B cells and cytotoxicity CD8+ T lymphocytes. Activation of the adaptive immune system requires antigen presentation on major histocompatibility complex (MHC) molecules and costimulatory signals [61].

Among antigen presenting cells, DCs are considered to be the most effective in inducing the adaptive immune response [62]. DCs exist in the thymus, spleen, and lymph nodes. Entrance into the lymphoid organs, are divided into two categories: 1. DCs developed from bone marrow precursor cells and travel through the blood to lymphoid organs; 2. DCs develop from precursor cells in peripheral tissues and after activation by pathogens, migrating through peripheral tissue to draining lymph nodes [42,43]. In the process of maturation, dendritic cells constantly express MHC-II molecules and costimulatory molecules, so as to activate specific immune cells and have strong migration ability. Therefore, dendritic cells are considered to be the major cells in the immune response. A subset of dendritic cells can also present exogenous antigens on MHC-I via cross presentation, leading to activation of CD8+ T cells [63,64].

In vivo, DCs have a unique ability to phagocytose pathogens without being infected themselves, and then go on to process, and present antigens from the pathogens to lymphocytes [65]. During these events, DCs undergo maturation. DCs present antigens in two ways: depending on whether the antigen is self or nonself derived. Peptides derived from intracellular proteins are displayed on the cell surface by binding MHC I molecules, which are specific for antigens. Whereas peptides produced by nonself containing proteins are displayed by MHC II molecules. When DCs are infected with viruses, the virus genome enters the nucleus and directs production of viral genes and proteins. The newly synthesized viral peptides are ubiquitinated, degraded by proteasomes, and the fragments are loaded onto newly formed MHC class I molecules. The MHC I/peptide complex is then transported to the cell surface for presentation, where CD8+ T cells respond by interacting with MHC class I bound antigen. In contrast, dead viruses engulfed by dendritic cells are degraded and trafficked in endosomes and then attached to MHC class II molecules for display on the cell surface [66,67],where CD4+ T cells recognize MHCII molecules and cause corresponding immune response. The maturation of dendritic cells is marked by MHC molecules, costimulatory molecules or co inhibition of expression, and the formation of more cytokines. After that, they entered the secondary lymphoid organ and activated T cells [68].

**Figure 2.**
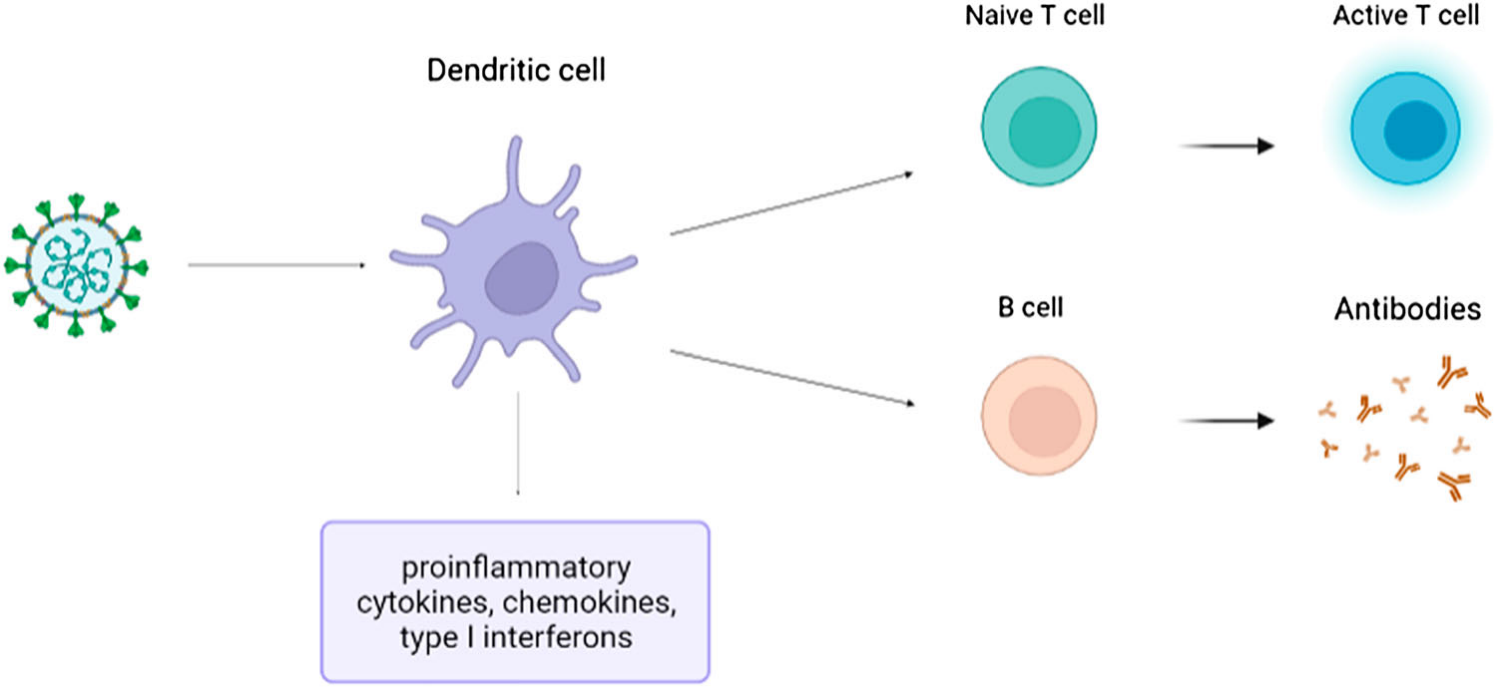
The role of dendritic cells in adaptive immune responses.

In addition, SARS-CoV-2 can effectively induce specific B cell response [69]. pDCs play a significant role in activating B cells and promoting their differentiation into plasma cells-α mediated process [70,71] (Figure 2).

## Infection of dendritic cells by SARS-CoV-2

In mildly infected lungs and upper respiratory tract, DCs are activated and induced to produce a positive immune response. To some extent, the number of activated DCs positively correlates with the severity of the disease. However, in severe infections, the number and maturation of DCs decrease, which is a sign of DC failure [72,73].

DCs are the key sentinel cells in organism. Previous works have indicated that DCs affects the process of MERS and SARS in the body. The coronavirus can spread through many cells, including the dendritic cells [74,75]. As mentioned earlier, when SARS-CoV-2 gets into the lung with air, it will invade cells with the help of ACE-2 expressed on the cell surface [5]. ACE-2 is also expressed in pulmonary interstitial DCs, indicating that DCs can be invaded by SARS-CoV-2 [76,77]. Furthermore, this possibility is increased due to microcellular proliferation or interaction with dipeptidyl peptidase (DPP) 4 receptor [76,78]. It is currently suspected that SARS-CoV-2 can also enter cells via DC-SIGN [79]. DC-SIGN is also expressed on DCs, which specifically recognizes viral proteins containing high mannose glycans [80]. Recently, studies have found a new infection pathway through the interaction of CD147 spike protein [81]. DCs also express CD147 [82], which further proves that SARS-CoV-2 has the possibility of infecting DCs.

The interaction between DCs and SARS-CoV-2 is still mysterious, but it may have some similarities with the typical SARS-CoV infection model. Fluorescence staining of SARS-CoV infected DCs reveal the early infection the microscope, where negative strands of viral RNA is tested in DCs, indicating that the virus can replicate within DCs. However, at later time points there is no increase in viral RNA. Additionally, through the detection of cytopathy, there is no increase in virus titre in the infected DCs or cell culture supernatant, which confirms that the virus replication is incomplete and abortion. DCs cannot produce infectious new viruses. Meanwhile, apoptosis related genes and maturation related genes in DCs are not induced. DCs show antiviral cytokine interferon α, interferon β, interferon γ and interleukin 12p40, proinflammatory cytokine (TNF-α And IL-6). This lack of antiviral cytokine response may be one of the mechanisms of SARS coronavirus evading immunity [65].

SARS-CoV-2 infection may lead to acute immune dysfunction. Clinical data showed that the percentage of dendritic cells in the peripheral blood was decreased in patients in the acute and convalescent phases (mean 13% and 30%) [83]. SARS-CoV-2 probably causes the cytopathy of DCs and reduces the number of dendritic cells. SARS-CoV-2 also decreases the function of DCs. Reported that CD11c+ conventional DCs from patients with acute coronavirus infection were unable to produce sufficient IFN γ – α And IFN-β [83]. In conventional DCs, expression of costimulatory molecules CD80 and CD86 is impaired. Consequently, proliferation of subsequently activated CD4+ and CD8+ T cells and adaptive T cell response to SARS-CoV-2 are also impaired [83–85]. The expression of HLA-DR in infected DCs decreased, indicating that the antigen presentation ability of DCs was also weakened [86]. Infected mo-DCs upregulated the expression of proinflammatory cytokines and chemokines and enhanced the severity of inflammation but not antiviral interferons and IFNs-α and IFN-β generation [65,87,88]. Thus, SARS-CoV-2 seems to escape from the body's immune mechanism by inhibiting the number of DCs and decreasing the function of dendritic cells and the number of IFN molecules produced [89], leading to impaired progression of innate and adaptive immunity. In critically ill patients using ventilator, DC is significantly reduced [90]. The failure of DCs may be part of innate antiviral immune activation in the acute phase of infection [91,92]. Furthermore, the rapid loss of DC numbers and function helps to explain the delayed T cell response and low level of IFN-γ/ IFN - III [50].

## Discussion

DCs not only play a part in the innate immune response, but also guide the subsequent adaptive immune response. There are still many unknown factors about the function of dendritic cells in SARS-CoV-2 infection. When DCs are exposed to virus, they are stimulated and develop into two different types. For one thing, cells that are not infected by the virus play an antiviral immune function, for another, cells that are infected by the virus participate in the process of immune evasion [77,93]. DCs are an important immune part for inducing adaptive immune response mediated by T cells and B cells. Furthermore, when pathogens are recognized, DCs can release a great quantity of cytokines and chemokines. Up to now, available data indicate that the acute infection of SARS-CoV-2 will result in the rapid consumption and abnormal function of host DCs. These functional abnormalities affect the outcomes of infectivity, clinical disease, and future re-exposure. Also, these DC changes impair immune memory, as well as vaccine efficacy. For therapeutic aspects, the number and function of DCs can be targeted to improve. Currently, modulation of dendritic cell maturation and thus immune response through mesenchymal stromal cells (MSCs) has been proposed as an alternative therapeutic strategy for COVID-19 [94].

Clinically, the number of female patients is lower than that of male patients. Does this mean that the probability of SARS-CoV-2 infection is lower in women than in men? Estrogen has a positive stimulating effect on dendritic cells, which is conducive to the activation of dendritic cells and lays a foundation for the generation of subsequent immune response. Furthermore, it has been shown that although two dendritic cell subsets, dendritic cells (DCs) and mucosal Langerhans cells (LCs), have not been infected by SARS-CoV-2, they are both able to efficiently capture virus through heparan sulfate proteoglycans and transmit the virus to ACE2 expressing cells, allowing them to become infected [95].

In the future, we need to continue to understand the interaction mechanism between SARS-CoV-2 and DCs in different stages of infection. Moreover, a comprehensive understanding will provide the knowledge for studying and creating new therapies [96].
